# Biomimetic Liquid Crystal Cilia and Flagella

**DOI:** 10.3390/polym14071384

**Published:** 2022-03-29

**Authors:** Roel J. H. van Raak, Dirk J. Broer

**Affiliations:** 1 Laboratory of Stimuli-Responsive Functional Materials and Devices, Department of Chemical Engineering and Chemistry, Eindhoven University of Technology, Groene Loper 5, 5612 AE Eindhoven, The Netherlands; r.j.h.v.raak@tue.nl; 2 Institute for Complex Molecular Systems, Eindhoven University of Technology, Groene Loper 5, 5612 AE Eindhoven, The Netherlands; 3 SCNU-TUE Joint Lab of Devices Integrated Responsive Materials, South China Normal University, Guangzhou Higher Education Mega Center, No. 378, West Waihuan Road, Guangzhou 510006, China

**Keywords:** artificial cilia, artificial flagella, biomimetic, liquid crystal polymer, liquid crystal elastomer, fibre drawing, additive manufacturing, replica moulding

## Abstract

Cilia and flagella are a vital part of many organisms. Protozoa such as paramecia rely on the collective and coordinated beating of tubular cilia or flagella for their transport, while mammals depend on the ciliated linings of their bronchia and female reproductive tracts for the continuity of breathing and reproduction, respectively. Over the years, man has attempted to mimic these natural cilia using synthetic materials such as elastomers doped with magnetic particles or light responsive liquid crystal networks. In this review, we will focus on the progress that has been made in mimicking natural cilia and flagella using liquid crystal polymers. We will discuss the progress that has been made in mimicking natural cilia and flagella with liquid crystal polymers using techniques such as fibre drawing, additive manufacturing, or replica moulding, where we will put additional focus on the emergence of asymmetrical and out-of-plane motions.

## 1. Introduction

Cilia and flagella are a vital part of many organisms [1]. In essence, a cilium and a flagellum are the same, though typically flagellated cells only contain one or a few flagella, while ciliated cells can be covered with many cilia [2]. Protozoa such as paramecia often rely on the collective and coordinated beating of tubular cilia or flagella for their transport. Similarly, a surface covered with active cilia is well-known to be able to transport cargo if the cilia beat collectively and coordinated [3]. This is, for instance, found in mammalian bronchia or fallopian tubes, where the cilia fulfil a crucial role in the continuity of breathing or reproduction, respectively. This unidirectional transport is made possible through an asymmetry in the cilium beating pattern that induces a unidirectional flow in the liquid medium surrounding the cilia [4]. Asymmetries can be distinguished in two categories: on the level of a single cilium (configurational asymmetry) [5], and on the cilium array level [6]. Configurational asymmetry arises when the tip of one cilium travels a non-reciprocal path with respect to the fluid flow [5], i.e., the cilium effective stroke is different from the recovery stroke [7]. The flow generated by the non-reciprocal beating of a single cilium is enhanced by the formation of metachronal surface waves over a ciliated surface [8].

The abundance of cilia and flagella in nature has inspired man to mimic these with synthetic materials, in an attempt to fabricate new and smart devices of continuously increasing autonomy and decreasing dimensions [4,9]. In recent years, several materials [9] have been used to mimic natural cilia and flagella, such as magnetically doped elastomers [10], or liquid crystal polymers. Magnetically responsive cilia are commonly prepared by blending a polymer precursor with magnetic particles, upon which the mixture is poured into a mould and allowed to set. After removing the mould, an array of polymer pillars is obtained that tracks a magnet moving nearby [11,12,13]. These magnetic cilia have successfully been used for marine antibiofouling [12], mass transport [11,14], or as (part of) a crawling robot [15]. Alternatively, by exchanging the magnetic particles for dielectric particles, a response to a changing electric field is also possible [16]. While this class of artificial cilia can, like a natural system, work untethered and, in some cases, also with some autonomy, bulky external equipment such as motorised magnets or function generators are required to be in close proximity for their operation.

**Figure 1:**
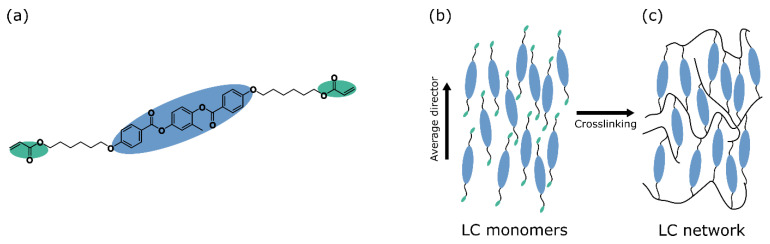
Preparation of LC polymers. (**a**) Typical molecular structure of a liquid crystal monomer, with the stiff core indicated in blue and the reactive end-groups, in this case acrylates, indicated in green. (**b**) A nematic mesophase of LC monomers with uniaxial order. The LC reactive end groups can be directly and densely crosslinked into an LC network (**c**), where the LC order is frozen in so that the mesophase is retained also when the material temperature is brought outside of the liquid crystal temperature window.

**Figure 2:**
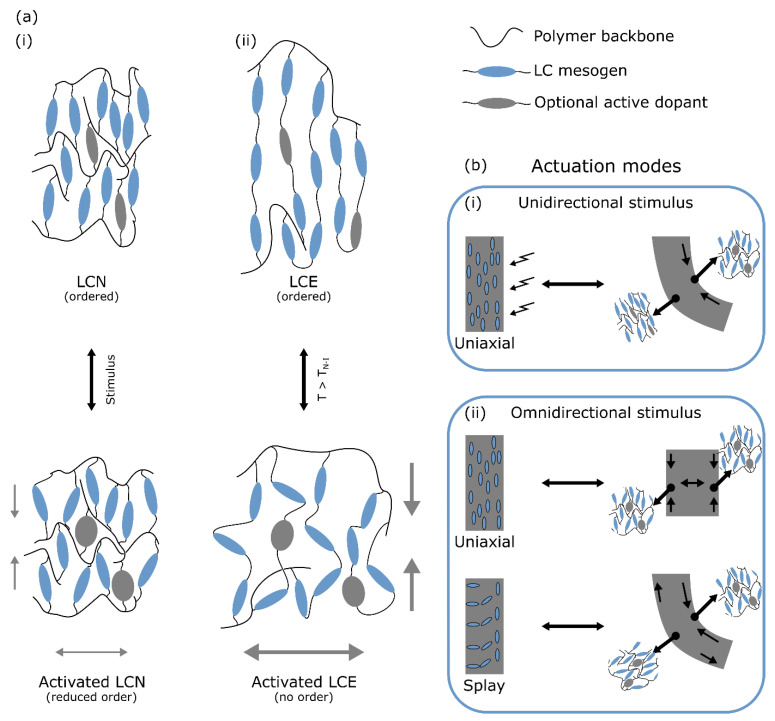
(**a**) Actuation mechanism of liquid crystal polymers on a molecular level triggered by a diversity of stimuli. (**i**) Homogeneously aligned liquid crystal networks show a uniaxial contraction along the alignment direction when disrupting the molecular order through an outside stimulus such as heat. The network expands in the directions orthogonal to the original LC director. Optionally, the network can be sensitised to other stimuli by incorporating active dopant molecules in the network structure. (**ii**) Liquid crystal elastomers are crosslinked less densely which makes a transition from the nematic to the isotropic phase possible. This reversible phase transition manifests as a strong contraction parallel to the molecular director and an expansion in the orthogonal directions. (**b**) Actuation of LC polymers on a device level. (**i**) Uniaxially aligned materials exposed to a stimulus on one side (e.g., one-sided light-illumination) will only activate on the exposed side if the active dopant concentration is appreciably high enough to create a gradient in stimulus absorption, causing one side to contract more than the other and thus inducing a bend in the material as if it were a bilayer of two materials with different coefficients of expansion. (**ii**) For omnidirectional stimuli such as heat, uniaxially aligned materials show a homogeneous contraction along the average LC director and an expansion in the orthogonal directions. Materials that have a gradient in alignment (such as splay) will always bend to a pre-programmed side regardless of stimulus direction.

As a material, liquid crystal polymers (LCPs) gained significant attention due to their responsiveness to a wide variety of environmental stimuli, while still retaining a considerable amount of mechanical strength. Of these stimuli, heat and light are the most common ones [17] due to the ease and accuracy with which they can be applied. LCPs can be prepared from liquid crystal monomer mixtures following techniques that are well-established for most monomer resins, which allows for a wide range of possible device shapes such as flat films, thin fibres, or micropillars. Thermotropic liquid crystal (LC) monomers are molecules characterised by one or more phases between the solid and liquid phase (the mesophases), and often have a rod-like shape (calamitic). The calamitic molecular structure is recognised as a stiff rod-like core that is supplied with flexible tails at one or both ends, where at least one end of the molecule is terminated in a polymerisable group (Figure 1a shows a typical diacrylate LC structure). In a mesophase prior to polymerisation, the molecules are able to flow like a liquid while maintaining some intermolecular ordering (like a crystal). The nematic mesophase is characterised by one-dimensional order where the long axes of the mesogens locally orient themselves in approximately the same direction along an average director (Figure 1b). In a smectic mesophase, the molecules’ long axes are not only oriented in a common direction but also show some positional order where the central molecular cores are confined to layers. The molecular orientation can be controlled over longer distances by mechanical stress-induced alignment, external field-induced alignment, and surface effect-induced alignment to obtain a uniform orientation of the average molecular director. The mesophase order is preserved by (photo) polymerising the reactive monomer end groups into an ordered (crosslinked) liquid crystal network (LCN, Figure 1c). Due to the elongated shape of the LC core, the polymer’s response to an external stimulus such as heat is not equal in all directions. As a result of the increased thermal motion and corresponding reduction in order upon an increase in temperature, the network shrinks along the average molecular director and expands in the orthogonal directions (Figure 2(ai)) [18]. The LC network can optionally be functionalised with active dopants such as azobenzene derived dyes, carboxylic acid salts, or nanoparticles to make the liquid crystal polymer actuate in response to light [19,20,21], water [22], or electricity respectively [23], as is outlined in Figure 2.

Most LCP actuators are prepared by cutting a small strip of material from an LCP film that was prepared in a glass cell treated with polymer alignment layers. In recent years, several new and existing processing methods have been employed for preparing (responsive) LCP devices on a variety of length scales such as replica moulding [24], fibre drawing [25], electrospinning [26], lithography [27], and additive techniques such as inkjet printing [28] or 3D-printing [29]. For fabricating artificial liquid crystal cilia and flagella, replica moulding, and fibre drawing are the most common ones as the shape of structures obtained through these methods can closely follow that of natural cilia/flagella. In this review, we will discuss the progress that has been made in mimicking natural cilia and flagella with LCPs, where we will put additional focus on the emergence of asymmetrical and out-of-plane motions.

## 2. Artificial Liquid Crystal Flagella: Active Drawn or Stretched Fibres

**Figure 3:**
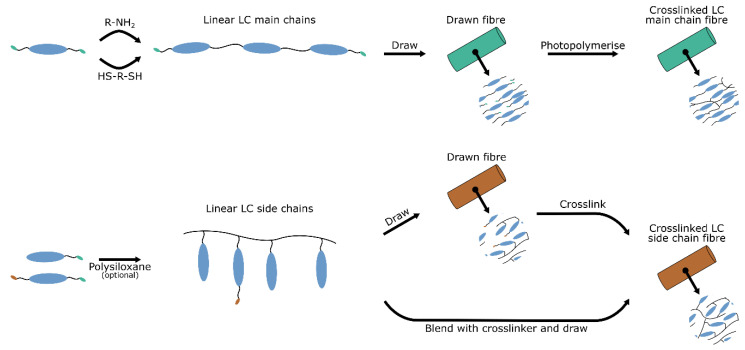
Schematic illustration of the preparation of main chain LC fibres (top scheme), and side chain LC fibres (bottom scheme). In both cases, LC monomers are extended into short (LC oligomer) or long (LC polymer) chains to increase the material viscosity. The viscous material is then drawn into a fibre and crosslinked to yield the final LC fibre.

LC fibres are most commonly drawn from a viscous mixture of liquid crystal oligomers or polymers, the drawn shape is fixed by a polymerisation process that takes place during [25] or (shortly) after [30,31,32] the drawing process. A high viscosity of the oligomer or polymer melt ensures a stable shape of the drawn fibre and is usually obtained by creating linear chains of conventional liquid crystal monomers containing several (LC oligomers) or many (linear LC polymers) repeating units. Common methods to obtain these linear chains are free radical polymerisation of mono (meth)acrylate liquid crystal monomers [25,33] or thiol-ene [34] or aza-Michael [35] click chemistry of diacrylate liquid crystal monomers using dithiols or primary amines as chain-extenders respectively. The linear LC chains all bear reactive groups so that the drawn fibre can be physically [25,34] or supramolecularly [36,37] crosslinked in a reaction orthogonal to the chain extension process. Shear generated by drawing aligns the oligomer or polymer backbone and mesogenic units along the fibre axis [38]. The molecular alignment in the fibre is finally frozen in together with the shape during the second crosslinking reaction [25]. The different pathways for obtaining crosslinked LCE fibres are schematically outlined in Figure 3.

**Figure 4:**
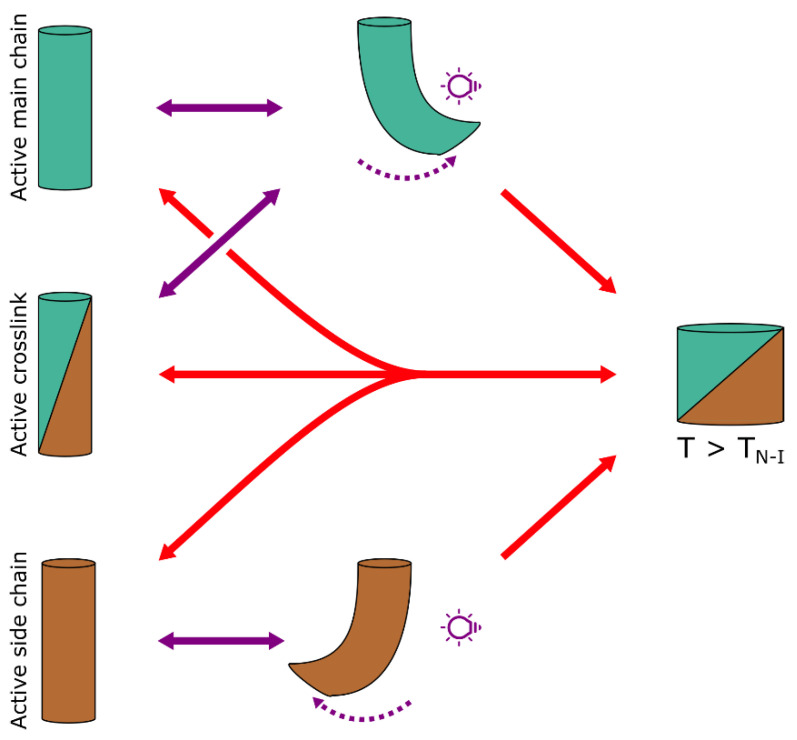
Schematic illustration of the different actuation modes of drawn LCE fibres. Fibres doped with a photomechanical chromophore in the main chain, or the crosslink will bend towards a (usually) UV-light source and return to their straight position with visible light illumination from any direction. Fibres that bear an azobenzene derivative pendant to the backbone bend away from a light stimulus. Additionally, heating the bent fibre can cause it to regain its straight shape though thermal relaxation of the chromophores. When the fibre temperature is appreciably high (above the nematic to isotropic transition temperature of the elastomer), the fibre will show a strong and reversible contraction along the fibre long axis.

The resulting elastomer fibres have a relatively low crosslinking density which leaves the LC mesogens with enough freedom to still pass through an isotropisation temperature (the T_N-I_) where the internal molecular order is lost. Due to the molecular alignment inside the drawn fibre, this translates into a strong yet reversible contraction along the fibre long axis once this temperature is crossed, schematically illustrated in Figure 4 [25]. Doping the fibre with photothermal agents such as certain nanomaterials [25,39] or azobenzene derivatives [40,41] makes these fibres responsive to specific wavelengths of light. The largest advantage is that the fibre temperature can now be controlled very locally due to the spatial and temporal accuracy of light as a stimulus. Recent work by Lin et al. has shown that this process of drawing LCE fibres can be easily scaled up by continuously drawing a fibre from the melt and crosslinking it by in-line UV illumination, before it is rolled onto a bobbin [34]. The authors have shown that by carefully controlling the drawing conditions, the mechanical properties of the fibre could be tuned and it retained its thermal response.

Palagi et al. have shown that by taking a small piece from a manually drawn and photocrosslinked fibre, they could create a self-propelling robot [41]. An azobenzene-derived dye that was bound covalently to the molecular backbone of the fibre converts light into heat to fuel a nematic to isotropic phase transition of the surrounding LC elastomer. By locally addressing this dye, the phase transition, and therefore expansion orthogonal to the fibre axis of the robot, could be accurately controlled along the length of the robot. Scanning the actinic light source over the length of the fibre created a traveling wave along the surface, propelling the robot forward, which resembles the peristaltic locomotion of worms or a metachronal surface wave as found on protozoa such as paramecia [41,42].

In case of the photothermal actuation mechanisms, the position of the chromophore in relation to the rest of the network is relatively unimportant, as it should primarily release heat to its environment. Unfortunately, most actuations that could be obtained by crossing the isotropisation temperature occurred along the fibre long axis due to the uniaxial molecular alignment (schematically outlined in Figure 2 and Figure 4), while we are looking for out-of-plane motion in order to provide a good cilium mimic. As a result of the uniaxial mesogen alignment in drawn LC fibres, the asymmetry that is required for an out-of-plane deformation needs to be introduced in another way. From work on homogeneously aligned LC strips, it is already well-established that increasing the concentration of, usually diacrylate functionalised, azobenzene derivatives copolymerised with a homogeneously aligned LC cantilever will create a strong gradient in light absorption through the film thickness [43]. Upon illuminating the cantilever from one side, the actinic light can only partially penetrate into the material, causing isomerisation events to selectively occur on the illuminated side, as illustrated in Figure 2(bi). This makes the material actuate as if it were a bilayer of a shrinking material (the illuminated side) and a stationary material (the “shadow” side), causing a bend towards the light source due to the mechanical pull a copolymerised azobenzene derivative exerts on the illuminated side of the network [44].

Liquid crystal fibres can also actuate out of plane following this mechanism. Depending on the position of the azobenzene derivative in relation to the rest of the elastomer network, the response of the surrounding material will be different, illustrated in Figure 4. Work on LC films has shown that azobenzene derivatives covalently bound to the network on only one side (as a pendant group) generally show a smaller response than cases where the azobenzene derivative is built into the crosslink and therefore connected to the network at both ends [45,46]. When used as a crosslinker, the azobenzene derivative can exert a much stronger pull on the network when isomerised into the *cis* isomer, maximising the disruption of the network, and accompanying deformation. In LC fibres, a third position can be considered, which is as part of the elastomer main chain. Liu et al. have varied the position of the azobenzene derivative in their manually drawn liquid crystal fibres and found that the bending in response to UV light in air and in water (where photothermal heating is eliminated) was strongest for fibres which had the azobenzene derivative placed in the main chain of the oligomer [47]. Increasing the crosslinking density by adding additional LC diacrylates reduced the response, while moving the azobenzene derivative from the main chain to the crosslink, reduced the response to UV light even further [47]. Another report by Cheng et al. investigated photoresponsive fibres in which the azobenzene was positioned as pendant group to the network. The authors found that, contrary to other reports where the azobenzene derivative is placed in the crosslink, the fibre would bend away from the actinic light source [48]. The authors postulate that the actuating mechanism for these fibres is different than other photomechanical mechanisms. Since the azobenzene isomerisation can no longer exert a pull on the network, the authors suggest that due to the increased width of the *cis* isomer, the illuminated fibre side expands slightly, inducing a bend away from the light source. Just like other photomechanical mechanisms, the actuation was fully reversible upon isomerising the azobenzene derivative back to its *trans* isomer.

**Figure 5:**
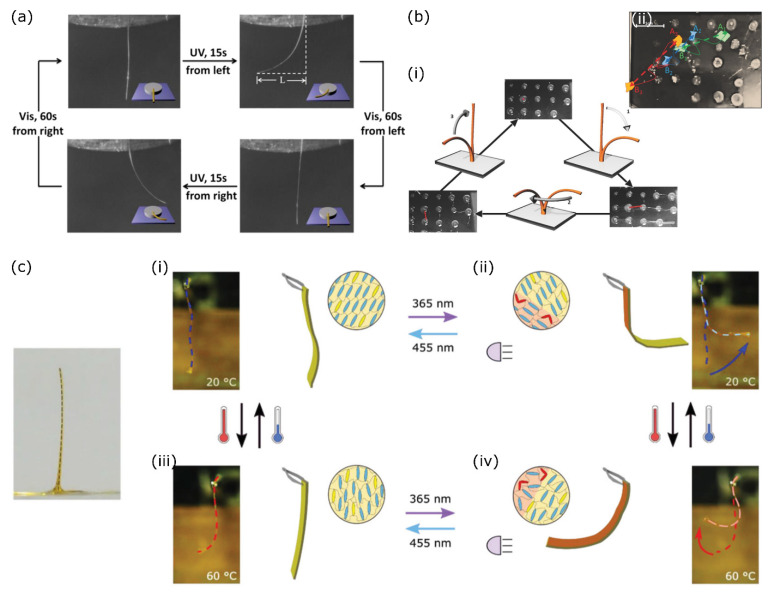
(**a**) Photoactuation of a heated and supramolecularly crosslinked LC fibre, illuminated from two directions with UV light. The fibre bends towards the UV light source regardless of direction and reverts to its straight orientation by visible light illumination. Adapted with permission from ref [36]. Copyright 2013 American Chemical Society. (**b**) Array of artificial LCE cilia that (**i**) exhibit an asymmetrical and collective beating motion by tracking a moving light source to (**ii**) transport cargo floating on top of the silicone oil surrounding the artificial cilia array. Adapted under the terms of the CC BY-NC Creative Commons Attribution-NonCommercial 4.0 International license [53]. Copyright 2016, The Authors. (**c**) Light responsive SmC-aligned polymer fibre. Illuminating a chemically identical LC cantilever with UV light at room temperature causes the cantilever to bend away from the light source and return with blue light illumination ((**i**) and (**ii**) respectively), at elevated temperature the cantilever is in the nematic state and shows opposite response: a bend towards a UV light source and a return upon blue light illumination ((**iii**) and (**iv**) respectively). Adapted under the terms of the CC BY Creative Commons Attribution 4.0 International license [30]. Copyright 2020, The Authors.

Work by the groups of Zhang [32,49,50] and Ikeda [51] demonstrated fibres made from copolymers of monoacrylate azobenzene-derived monomers, some of which were supplied with a functional group that could be used for crosslinking the fibres. The copolymers were melted and drawn into a fibre by pulling some material with a pair of tweezers from the melt. The linear polymer chains were interconnected at the reactive azobenzene sites by introducing a crosslinking agent either during fibre drawing [51] or post drawing by immersing the fibre into a crosslinker solution [32]. In the resulting fibres, the azobenzene derivative is placed in the crosslink. When the azobenzene derivatives are thiol-terminated, the drawn fibres can be crosslinked and de-crosslinked by immersing them in an oxidiser or reducer solution, respectively forming (or breaking) disulfide bonds between the polymer chains [50]. This allows the fibres to be recycled into linear polymers that could later be used to fabricate new fibres with similar responsiveness as the original fibres. Alternatively, the azobenzene-doped fibres can be crosslinked through supramolecular interactions such as hydrogen bonds, as was demonstrated by the group of Zhang [36,37]. The mechanical rigidity provided by the hydrogen bonds ensures these polymer fibres are similar in strength to the chemically crosslinked ones. In all cases, the crosslinked fibres bent towards an actinic UV light source of high intensity, but only if the fibres were heated above their glass transition temperature. A bent fibre returned to its original straight position by visible light illumination from any direction, as shown in Figure 5a. Moving the UV light source in relation to the fibre caused the fibre to bend towards the new position of the light, demonstrating their ability to track a light source. Liu et al. have developed a similar system based on azotolanes instead of azobenzenes, with the main advantage being that the material can now be fully addressed with visible light instead of harmful UV light [52]. The photogenerated stresses parallel to the fibre axis were on the same order as human muscles [51,52], underlining the potential application of these materials as light-driven artificial muscles.

By bringing together a set of liquid crystal fibres attached to a surface, Gélébart et al. have shown to be able to create a cilia-like fibre array that could be fully addressed by light [53]. Immersing the fibre array in warm silicone oil made sure that the fibres could be addressed with, and track, a single light source since the elevated temperature ensured appreciably fast thermal *cis/trans* relaxation of the azobenzene derivative. By moving the UV light source, the fibre array could be made to move in an asymmetric, coordinated, and concerted motion, as schematically shown in Figure 5(bi). The upright fibres were illuminated with UV light from one side, causing them to bend forward. Rotating the light source 90° around the sample caused the fibre to follow this rotation. The initially upright shape was regained by illuminating the fibre from the top so the actuation process could be restarted. Due to the collective motion, a flow was created in the oil that was strong enough to move some particles floating on top of it, strongly resembling transport induced by ciliated surfaces (Figure 5(bii)) [53].

More recent work by del Pozo and Liu et al. described a main chain azobenzene-doped liquid crystal oligomer [30]. The oligomer was suitable for being drawn into a self-standing fibre (Figure 5c) but could also be 3D-printed into actuator strips. After photopolymerisation, these strips had a smectic C (SmC) alignment at room temperature, which, upon isomerisation of the incorporated azobenzene derivative, could transition into a smectic A (SmA) alignment, shown in Figure 5(ci,cii). Unlike what is observed in most light-responsive LC polymers, this caused a bend away from the UV light source as the SmC-SmA phase transition is accompanied by a small expansion along the average molecular director. When heating the fibre to the nematic phase, the UV response inverted and a reversible bend towards the actinic light source was observed as is the case with most other nematic elastomer fibres (Figure 5(ciii,civ)). Increasing the temperature further brought the elastomer fibre into the isotropic phase, causing a strong contraction along the fibre long axis [30]. The fibre retained its versatile responsiveness when submerged in water.

## 3. Artificial Liquid Crystal Cilia: Reducing Size and Spacing

Natural cilia and flagella have a width of only several hundreds of nanometres and a length typically ranging from 2–15 µm [1] to over 2 mm in some cases [2]. These sizes are challenging to obtain through the (manual) fibre drawing methods discussed above, so in order to obtain a reduction in feature size different processing techniques are considered. A large reduction in feature size can be obtained by using additive manufacturing techniques such as inkjet or 3D-printing, or 3D photolithography.

**Figure 6:**
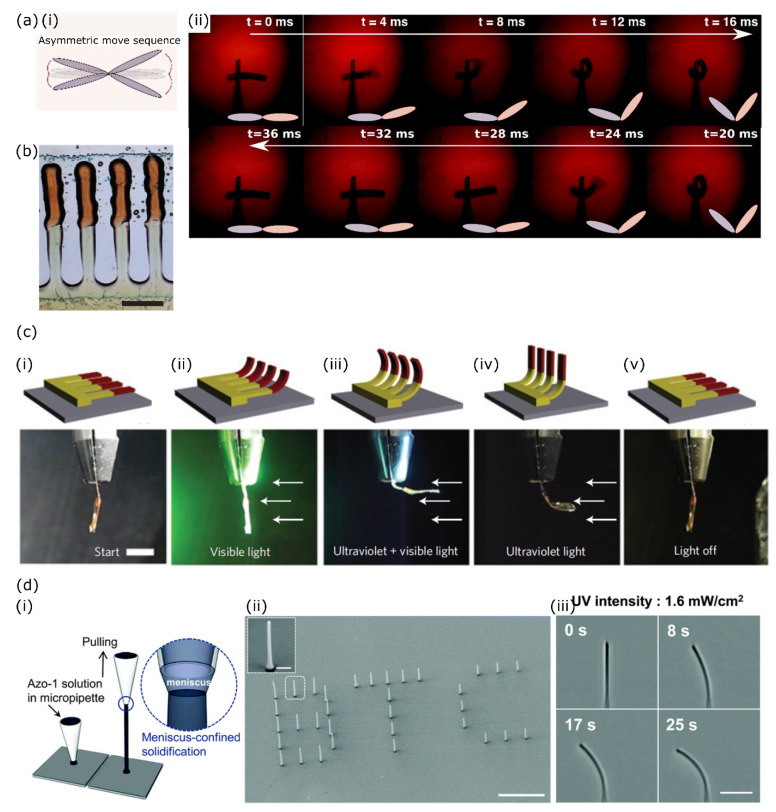
Responsive liquid crystal microstructures obtained through additive manufacturing techniques. (**a**) (**i**). Schematic illustration of the composite microactuator obtained through 2PP combined with DLW. (**ii**). Frames obtained from a high-speed video of the composite microactuator bending asymmetrically in response to a homogeneous stimulus. Adapted under the terms of the CC BY Creative Commons Attribution 3.0 Unported license [55]. Copyright 2017, The Royal Society of Chemistry. (**b**) Inkjet-printed artificial composite cilia and (**c**) their actuation sequence. The cilia at rest (**i**) are illuminated with visible light to address one azobenzene derivative (**ii**), adding UV light addresses both azobenzene derivatives (**iii**). Removing the visible light allows one azobenzene derivative to relax (**iv**). The cilia return to their rest state when all light is switched off (**v**). The scale bar of b is 0.5 mm and the one of c 5 mm. b and c are adapted from ref [28]. Copyright 2009, Nature Publishing Group. (**d**) (**i**). Schematic of the 3D deposition technique used to fabricate self-assembled light-responsive fibres. (**ii**). SEM micrograph of fibre arrays where the three letters consist of fibres of different lengths (L to R: 20, 15, 10 µm), demonstrating the controllability and versatility of the approach. (**iii**). Optical micrographs of a UV-illuminated fibre over time. The UV light is incident from the left. Adapted under the terms of the CC BY-NC Creative Commons Attribution-NonCommercial 3.0 Unported license [56]. Copyright 2015, The Royal Society of Chemistry.

By using two-photon polymerisation (2PP) combined with direct laser writing (DLW), the group of Parmeggiani has developed a method that allows them to “3D-write” responsive liquid crystal structures with a submicrometer resolution. A cell is filled with a reactive LC monomer mixture, which is in turn locally illuminated by a focused pulsed laser with a wavelength of twice the photoinitiator absorption wavelength. In the focal point of the laser, the photoinitiator is able absorb two photons simultaneously, which makes the initiation occur very locally in 3D space. By moving the laser focal point across the cell, 3D structures can be written from a liquid precursor with high accuracy. Any unreacted material is washed away after the DLW, making this method material and time intensive. If the monomeric LC is aligned in the cell, the alignment in the polymerised structures is preserved [54]. In a later publication, the authors demonstrate a method of using their 2PP writing process to make composite microactuators. They first create structures of one liquid crystal monomer mixture, open the cell and remove any non-reacted components. Using the structured substrate to fabricate a second cell and filling this with new monomer material, the authors have shown that they can incorporate two different LC mixtures in the same microstructure, and hence also obtain two different responses to the same stimulus (light in their case, schematically shown in Figure 6(ai)). This made it possible for them to generate the asymmetrical motion shown in Figure 6(aii), realising a promising next step towards mimicking the motion of natural cilia with a single stimulus [55].

Alternatively, by using a printing technique, the manufacturing time and material losses can be reduced as was demonstrated by Van Oosten et al. [28] By inkjet printing two different solutions of LC monomers, each containing a different azobenzene derivative, the authors create a linear array of artificial cilia with an asymmetric response to light. The base and tip of the cilia contain different azobenzene-derived dyes, an array of printed composite cilia is shown in Figure 6b, and therefore bend in response to different wavelengths of light making them selectively addressable. Following a specific illumination sequence, the printed artificial cilia could be made to actuate underwater where the forward stroke was different from the backward stroke, strongly resembling the beating pattern of natural cilia [28]. This illumination sequence is outlined in Figure 6c. Visible light illumination addresses the azobenzene derivative in the cilium tips (the clamped side in the lower row of pictures), adding UV light addresses both azobenzene derivatives in the actuator, causing the whole cilium to bend (Figure 6(ciii)). Removing first the visible light and then the UV light causes the cilium tips and base to relax to their initial position respectively (Figure 6(civ–cv)).

Another example comes from Lee et al., which nicely shows a method for decorating a surface with LC pillars several microns in height. The authors prepared a solution of an azobenzene-derived dye which forms supramolecular columnar stacks with itself and loaded this in a micropipette. Depositing some solution on a substrate and gently pulling the pipette away from it allows a supramolecular fibre to form with a width of approximately 680 nm and a controllable height of up to 20 µm (see Figure 6(di,dii)). The resulting fibres bend towards a UV light source of low intensity (Figure 6(diii)), making the device suitable for application in biological environments [56].

## 4. Arrays of Artificial Cilia: Increasing the Number Densities

To fabricate (micro)structures through replica moulding, a malleable (monomer) material is introduced in a structured mould or template with a negative imprint of the desired structures. The materials’ shape is fixed by curing it inside the mould, which in turn can be removed by either peeling it off [57] or dissolving it in a non-solvent for the structures [58]. Translating replica moulding onto liquid crystal technology has as advantage that the mould, provided that the negative structures are appreciably small, controls the molecular alignment by confined ordering mechanisms employing the elasticity of the LC. When LC monomers in their LC phase are confined to an anisotropic space smaller than the average LC domain size, the average LC molecular director will adapt itself to the shape of the space so that the director is oriented parallel to the long axis of the space the LC is filling [58,59]. These confinement effects are still relatively weak, which allows for realignment of the LC director by other external fields such as alignment layers on the template [60], electric [61], or magnetic fields [62].

Replica moulding allows for easy fabrication of large numbers of structures in a single process step, where the structure size and shape are highly tuneable and directly related to the mould. These moulds are often made through straightforward lithographic techniques using materials like PDMS [63] or silicon [64] so that the position and spacing of the individual structures can be accurately controlled. Alternatively, a track-etched membrane can be used as template. In this case the spacing and position of the individual features is less controlled, owing to the random nature of the tracking process, though track-etched pores generally allow for smaller feature sizes to be fabricated in high number densities [58,61].

**Figure 7:**
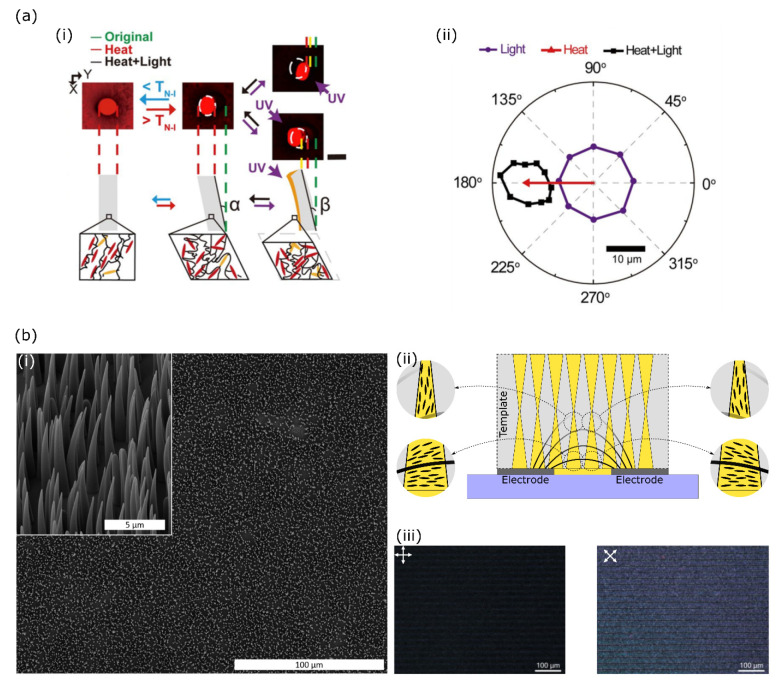
(**a**) Multiresponsive LCE micropillars. The LC alignment in each pillar is controlled by an external magnet during polymerisation of the LC mixture and programs a temperature-triggered actuation direction into each pillar ((**i**), left and middle). Shining a UV light on any side of the (thermally actuated) pillar causes a bend in the direction of the light-source ((**i**), middle and right). The LCE pillars can actuate in response to light or heat, and to a combination of the two, potentially introducing asymmetry in the actuation pattern (**ii**). Adapted with kind permission from ref [62]. Copyright 2018, The Authors. (**b**) SEM micrograph of microsized, tapered LC cilia as observed from above (**i**) and at a tilt of 45° ((**i**), inset). An electric field, as schematically indicated by equipotential field lines, realigned the LC monomers prior to- and during polymerisation so that symmetry in alignment was broken and the actuation direction was programmed into the polymer cilia, which is always perpendicular to the electrode lanes (**ii**). Polarised microscopy confirmed that the LC director in the self-standing cilia could be harnessed over large areas and conformed to the expected orientation shown in ii (**iii**). Adapted from ref [61]. Copyright 2022, Eindhoven University of Technology.

Yao et al. have for the first time demonstrated a method of actuating LCE pillars out of plane [62]. Inspired by the work of Keller [57] they filled a PDMS mould with polymerisable photoresponsive LC material and aligned this in a magnetic field. By reorienting the magnets with respect to the sample, the alignment inside the LC pillars could be changed. The obtained LC pillars bend towards a pre-programmed side upon the nematic to isotropic thermal transition, as dictated by the orientation of the sample with respect to the magnetic field during polymerisation (Figure 7(ai), middle) [62]. On top of this thermal response, the pillars also showed a light-tracking photoresponse due to the incorporated azobenzene derivative, in both the nematic and isotropic phases of the LCE pillars (Figure 7(aii)). In a later publication, the authors expanded their platform of LC micropillars to also preprogram the actuation behaviour in response to light. By carefully controlling the direction of the magnetic field present during polymerisation, and therefore the nematic director, microstructures could be fabricated that bent towards or away from the light source or twisted along their long axis upon (UV) light illumination [65]. The versatile and orthogonal responses of the fabricated microstructures to the two reported stimuli potentially allows for asymmetric beating patterns of individual pillars if the preparation and actuation conditions are carefully chosen.

By replicating hourglass-shaped pores from a track-etched polyimide template using a photoresponsive LC mixture, we have recently demonstrated a new platform of microsized tapered LC cilia [61] shown in Figure 7(bi). Through this approach, millions of LC microcilia per square centimetre could be prepared in a single polymerisation step. The presence of an electric field during polymerisation of the LC mixture controls the alignment of the LC mesogens inside the cilia. When the field was applied through surface-patterned interdigitated electrodes, the electric field direction, and therefore the local liquid crystal director, varied spatially as determined by the underlying electrode structure and laterally as determined by the distance from the electrode patterned substrate. For most polymer cilia, this caused symmetry to be broken within one cilium and the LC to be splay aligned, schematically demonstrated in Figure 7(bii) for two different cilia placed between two electrodes. Through polarised microscopy we confirmed that the LC director in the self-standing microcilia could be controlled over large areas (Figure 7(biii)). Increasing ambient temperature or illuminating the cilia array with UV light from any direction caused the cilia to reversibly actuate and bend towards a preprogrammed side as determined by the underlying electrode pattern. Like natural cilia, our artificial cilia array could be made to beat continuously by constantly shining blue light and switching a UV light on and off with a frequency matching that of the desired cilium beat [61]. The dimensions of these cilia were similar to those found in human bronchia, showed high number densities, and were homogeneously distributed over large areas.

## 5. Artificial Liquid Crystal Cilia: Remaining Challenges

There has been great progress in mimicking natural cilia and flagella with liquid crystal polymers, though some hurdles remain. Cilia are well-known for their autonomous and asymmetric motion, which, so far, has not yet been fully obtained in liquid crystal polymers. The amount of liquid a cilium displaces is maximised when the area is enclosed by the moving cilium tip (the actuation envelope) is largest [66]. The softness of LCE fibres allows for large, sometimes asymmetrical, out-of-plane deformations, where the fibre tip can describe a large area. The sizes in which these fibres are currently prepared are not yet on the order of cilium or flagellum length-scales. Conventional fibre-drawing techniques do not allow for fibres to be prepared on a micrometre size, so alternative approaches should be considered if the dimensions of drawn LCE fibres are to be scaled down. One promising method is the deposition of linear LC (oligomer) chains through a micropipette, where each microfibre is individually placed, drawn, and polymerised on a surface to eventually create a responsive array. While there is great control over the array layout and fibre dimensions, the fabrication process will have limited control over internal LC alignment and could be extremely time-consuming as even a single paramecium can be covered in 4000 motile cilia [67].

Through replica-moulding techniques, large numbers of active artificial cilia can be prepared in a single processing step. We have seen that high number densities of cilia can be obtained in this way and their dimensions can closely follow those of natural cilia, but that the actuation magnitude of these artificial cilia is still fairly modest. This is partially due to the stiffness of the material that is required to obtain closely packed self-standing structures. Soft structures will bend more, but also irreversibly stick to neighbouring structures should they come into contact [68]. Moving the artificial cilia from air to a liquid environment or increasing the spacing between two cilia could potentially circumvent this problem. Secondly, the microstructures are prepared in a mould which must be removed after curing of the infiltrating material. In order for the newly fabricated microstructures to survive the demoulding process, some mechanical rigidity is required. This can be obtained by reducing the aspect ratio of the artificial cilia and the surface area with which they contact the mould, though this does limit the potential magnitude of the actuation. Alternatively, a sacrificial layer can be applied between the mould and the microstructures, or the mould itself can be sacrificed after the replication process, bypassing the demoulding step. The latter approach has already been successfully used to obtain a suspension of responsive rod-like liquid crystal nanoactuators [58]. The formation of stable cilia-like structures obtained through replica-moulding ultimately depends on a careful balance in the material properties, mould design, and fabrication process.

The challenge of obtaining an asymmetrical response from LC polymer cilia has not been completely solved yet. Some examples rely on the sequential application of two orthogonal stimuli to obtain asymmetrical actuation. The artificial cilium can be composed of two parts where each individual part responds to one of the stimuli, other reports described cilia with an (orthogonal) response to both stimuli built into the same device. To keep the device simple, the asymmetrical response ideally arises from a single stimulus, as is the case with most photoresponsive drawn LC fibres. They rely on the directionality of the incoming light to introduce the asymmetry in the motion pattern, since the fibre itself is homogeneous in its composition and response. If the stimulus source moves asymmetrically, so will the fibre, though this makes the actuation process quite cumbersome. Programming asymmetry into the fibres themselves requires the presence of a gradient in either composition, alignment, or material properties inside the material. Such a requirement complicates the fabrication procedure and requires additional processing steps such as fibre rotation [40,69] to be carried out.

Since light is the most common stimulus for artificial cilia due to its accuracy and ease of administration, it should be able to reach all the structures. Especially in devices where the artificial cilia are densely packed on a surface, this can become a problem, as cilia can be shadowed by their neighbours. Changing the illumination conditions or direction provides a potential solution, as could changing the stimulus. Each stimulus does come with its own considerable drawbacks, so this is not preferred: magnetic fields rely on a nearby moving magnet; electricity is carried to the device through wires; heating the device is usually performed in an oven and has therefore slow response times.

In conclusion, the ideal mimic of natural cilia and flagella using liquid crystal polymers does not yet exist, as some conflicting choices have to be made in the design. Natural flagella are, due to their individual nature, best described by soft drawn LCE fibres that undergo large deformations. Though incorporating asymmetry in these fibres for sustained unidirectional locomotion is currently only achieved by complicated illumination setups, featuring several (moving) sources of light. Changing to a fabrication method based on small molecules rather than longer oligomers allows for more control over the internal molecular alignment, and therefore the built-in bending direction. Unfortunately, this likely sacrifices a large part of the actuation envelope as the resulting LCN material will be considerably stiffer due to the higher crosslinking density typically found in LCNs. Preparing arrays of artificial cilia, most commonly through replica moulding, faces similar challenges, with the addition of finding a balance between surface coverage and motility. Using replication techniques, the control over internal molecular alignment and beating patterns is good. Densely packed artificial cilia can unfortunately only be made of a harder, densely crosslinked polymer, so that the stickiness of the cilia is minimised. Consequently, the actuation envelope will be fairly limited due to the materials hardness. With increased spacing, a softer and more motile material can be used. The additional advantage is that applying a (light) stimulus reaches all cilia more easily, potentially enabling more uniform beating patterns.

## Data Availability

The data presented in this review article can be obtained from the referenced sources.
